# Isolation and Molecular Identification of *Leishmania* spp. Agents in Patients with Cutaneous Leishmaniasis in Yazd Province, Endemic Region of Central Iran

**Published:** 2020-05

**Authors:** Gilda ESLAMI, Ali FATTAHI BAFGHI, Mohammad Hassan LOTFI, Farzaneh MIRZAEI, Somayeh AHMADI, Ali Akbar TAJFIROUZEH, Hamid JAFARIZADEH, Sayyed Alireza PORMAZAR, Mahmoud VAKILI

**Affiliations:** 1. Department of Medical Parasitology and Mycology, School of Medicine, Shahid Sadoughi University of medical Sciences, Yazd, Iran; 2. Deputy of Health, Health System Research Unit, Shahid Sadoughi University of Medical Sciences, Yazd, Iran; 3. Department of Social Medicine, School of Medicine, Shahid Sadoughi University of Medical Sciences, Yazd, Iran

**Keywords:** Cutaneous leishmaniasis, Iran

## Abstract

**Background:**

Cutaneous Leishmaniasis (CL) is a major health problem in many parts of Iran. Many methods have been introduced for detection and identification of Cutaneous Leishmaniasis. The purpose of this study was isolation and molecular identification of *Leishmania* spp. agents in patients with CL from endemic region of central Iran. In this study, one of the main loci of central Iran named Yazd will be assessed CL identification using PCR-RFLP.

**Methods:**

For this cross-sectional study, sampling was done from 372 suspicious patients with CL who referred to Health Centers of Yazd Province from 2016 to 2017. After collection samples of patients, DNA extraction was done from samples on slides. Genus detection was done using specific primers by PCR. RFLP analysis was done for species identification.

**Results:**

Out of 372 samples, 159 samples were positive using PCR based method. Out of 159 samples, 87 (54.7%) *L. major* and 72 (45.3%) *L. tropica* were identified using RFLP analysis. The number of lesions in each patient was different but 119 (74.8%) patients showed the number of 1–3 lesions, and more lesions (more than 10 lesions) was showed in 4 (2.5%) person.

**Conclusion:**

The CL found in Yazd province resulted from *L. major* and *L. tropica* as the agents of rural and urban types, respectively. The prevalence of L. major and *L. tropica* was almost the same*.* This indicated that control programs could be designed for treatment and vector and reservoir control.

## Introduction

Leishmaniasis is a multi-form disease that causes by *Leishmania* spp. with distribution in more than 98 countries and affecting 1.5 to 2 million cases, annually (1). These multi-form diseases include self-limited Cutaneous Leishmaniasis (CL), Muco-Cutaneous Leishmaniasis (MCL), Visceral Leishmaniasis (VL) or Kala-Azar, and diffuse Cutaneous Leishmaniasis (DCL). CL is the common form with *L. major, L. tropica,* and *L. aethiopica* as the main agents in Old World (2). The CL distrusted in Iran is caused by *L. major* and *L. tropica* that named Zoonotic Cutaneous Leishmaniasis (ZCL) and Anthroponotic Cutaneous Leishmaniasis (ACL), respectively (3–7). The common diagnosis of Leishmaniasis is based on microscopic visualization of Giemsa-stained smears and culturing in monophonic and biphasic culture media such as NNN and RPMI 1640, respectively. These methods detect the genus of *Leishmania*, but in order to species identification, the higher sensitive and specific tools are needed. The molecular approaches are used for molecular detection and identification of many protozoan’s (8, 9). On the other hand, diagnosis of CL is difficult in the atypical forms such as ecthyma, tuberculosis, furuncle, ecthyma, atypical mycobacterium infections, deep mycosis, Sarcoidosis, leprosy, syphilis, foreign body granuloma and even sometimes malignant skin tumors. PCR based techniques are considered as a suitable tool with high sensitivity and specificity for *Leishmania* detection and identification (10). Molecular detection and identification of *Leishmania* spp. has been reported by many scientists (6, 8, 11).

The purpose of this study was isolation and molecular identification of *Leishmania* spp. agents in patients with CL from endemic region of central Iran. We detected and identified *Leishmania* isolates obtained from patients with suspicious CL referred to Health Center of one of the important endemic area of Yazd Province as the central one in Iran.

## Materials and Methods

**Fig. 1:**
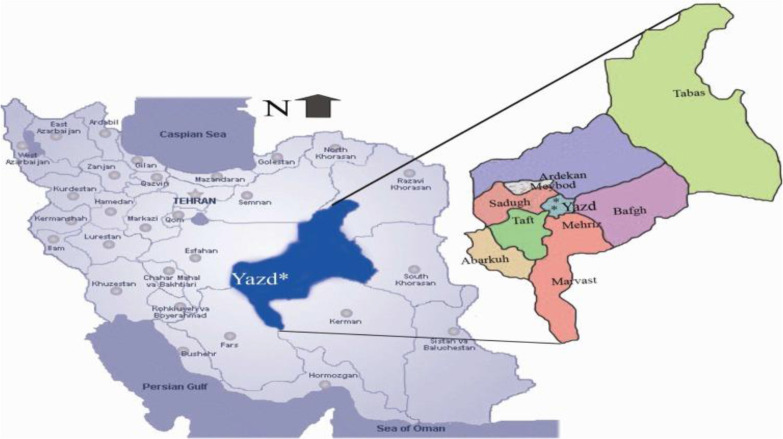
Map of Iran indicating the location of Yazd County situated in the center of Yazd Province (29). Available from: https://www.researchgate.net/figure/Map-of-Iran-indicating-the-location-of-Yazd-County-situated-in-the-center-of-Yazd-Province_fig1_229162444 (accessed 19 Jan, 2017).

Yazd Province was considered as the area for sampling. The capital of this province is named Yazd located the central of Iran with a population of 1,074,428. The climate of Yazd is dry with blazing sunshine and no humidity with average rainfall of around 60 mm, annually. It contains 20 cities including Abarbarkuh, Ahmadabad, Aghda, Ardakan, Ashkezar, Bafgh, Behabad, Hamidiya, Herat, Khezr Abad, Marvast, Mehrdasht, Mehriz, Meibod, Nadushan, Nir, Shahediyeh, Taft, Yazd, Zarach (Fig. 1).

### Patients

For this cross-sectional study, 372 patients with skin diseases and suspicious of CL who referred to Health centers of Yazd during 2016–2017, were investigated in this study. The patients lived in one the cities of Yazd, Abarbarkuh, Ardakan, Bafgh, Khatam, Taft, Mehriz, Ashkezar, and Meibod. Each patient included in this study completed the consent form and was interviewed for completing the data sheet containing the demographic data and other data including, the number of lesion, duration of disease, any treatment, and so on.

### Ethics

This project has the Research Ethics Committee code of IR.SSU.REC.1394.13 from Shahid Sadoughi University of Medical Sciences, Yazd, Iran.

### Sample collection

After cleaning and sterilizing of the skin using 70° ethylic alcohol, sample collection was done by scrapping of the margin lesion. Samples were used for examination slide and DNA extraction. From each patient, two slides were prepared, one slide for DNA extraction, and one for storing.

### DNA extraction

For DNA extraction using the DNA Gen All Exgene Cell SV (#106-101, Gen All, Korea) as manufacture’s instruction. The extracted DNA was analyzed in quality and qualification, using spectrophotometer and agarose gel electrophoresis, respectively.

### Detection and Identification

Genus detection was done using ITS1-PCR by the specific primers of LITSR (5′-CTG GAT CAT TTT CCG ATG- 3′) and L58S (5′-TGA TAC CAC TTA TCG CAC TT-3′). The reaction PCR included the final concentration of 1x PCR buffer, 0.5 nM each primer, 0.2 mM dNTP, 1.5 mM MgCl2, and 5 mM DNA. The condition of amplification was done by first denaturation of 94 °C and followed by 40 cycles of denaturation at 94 °C, annealing at 50 °C, and extension at 72 °C; each in 45 sec. The final extension was done at 72 °C for 5 min. The PCR product was assessed using 1% agarose gel electrophoresis alongside with 50 bp DNA ladder. The species identification was performed using RFLP analysis using *HaeIII* restriction enzyme with the concentration of 10 U in each reaction of 20 μl volume. The digestive assessment was done using 3% agarose gel electrophoresis alongside with 50 bp DNA ladder.

### Statistical analysis

Statistical analysis was done using SPSS ver. 16.0 (Chicago, IL, USA) by Chi-Square and significant differences of *P*-value<0.05.

## Results

Out of 372 suspicious patients in this study, 244 (65.6%) were male, 128 (34.4%) were female. Out of 372 samples, the highest and the lowest samples studied were from Yazd and Ashkezar/Mehriz with 249 (66.9%) and 6 (1.6%), respectively.

**Fig. 2:**
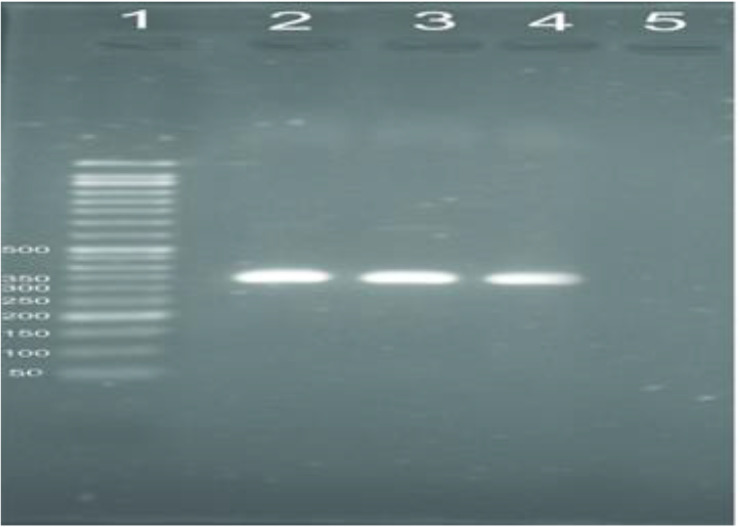
Molecular detection of *Leishmania* species. Lane1: 50 bp DNA ladder; Lane 2: *Leishmania major* (MRHO/IR/75/ER); Lane 3: *Leishmania tropica* (MHOM/IR/NADIM3); Lane 4: unknown sample detected as *Leishmania* spp.; Lane 5: negative control

**Fig. 3:**
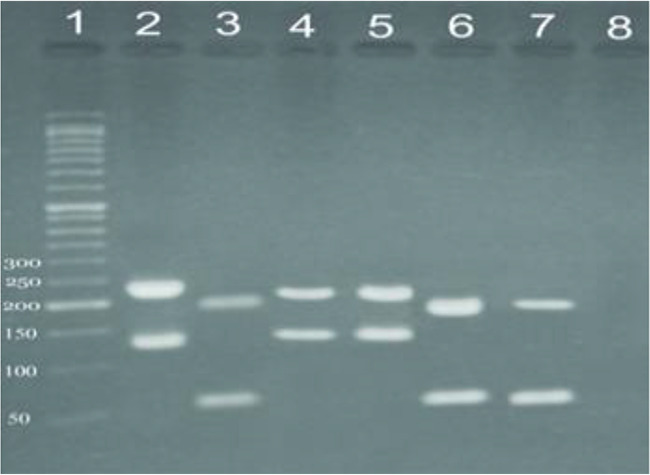
Molecular identification of *Leishmania*. Lane 1: 50 bp DNA ladder; Lane 2: *Leishmania major* (MRHO/IR/75/ER); Lane 3: *Leishmania tropica* (MHOM/IR/NADIM3); Lanes 4–5: unknown sample detected as *Leishmania L. major*; lanes 6–7: unknown sample detected as *L. tropica*; lane 8: negative control

Out of 372 samples, 159 samples were positive using PCR based method (Fig. 1). Out of 159 samples, 87 (54.7%) *L. major* and 72 (45.3%) *L. tropica* were identified using RFLP analysis (Figs. 2, 3). The number of lesions in each patient was different but 119 (74.8%) patients showed the number of 1–3 lesions, and more lesions (more than 10 lesions) was showed in 4 (2.5%) people. RFLP analysis with pattern of 220 and 140 bp fragments was considered as *L. major* and fragments of 200 and 80 bp was identified as *L. tropica*.

## Discussion

Leishmaniasis is one the important diseases that many researchers have been done in various aspects (1). The maintenance of this disease has been reported from various parts of the world due to immigration, population growth, presence of infected *Phlebotomus* spp., and suitable environment (12). In a retrospective analysis of clinical data from individuals visited health centers of Yazd from 2009 to 2013 showed that 897 people were infected with CL including 457 males and 439 females that these results revealed that the prevalence of disease in Yazd is high.

Various methods have been provided for Leishmaniasis detection identification but PCR based techniques have been considered as one the sensitive and specific method (13–15). This kind of method has been performed for Leishmaniasis identification in Iran (16–19). Molecular identification of different species including, *L. major* and *L. tropica* in Yazd as the major endemic area in Iran was the main goal of this study. In this study, out of 372 samples, 159 samples were positive using PCR based method. Out of 159 samples, 87 (54.7%) *L. major* and 72 (45.3%) *L. tropica* were identified. Recently, some researchers showed the same incidence of *L. tropica* and *L. major* among patients with CL (5, 8). In this study, the frequency of these two main species in this region was almost the same. Even though the majority of *L. major* reported from rural area but *L. tropica* detected from urban area such as Yazd City. These data are agreement with other studies in Iran (20, 21).

The majority of *L. major* was identified from Bafgh, Ardakan, Meibod, and Taft. Our study showed that more prevalence of Leishmaniasis is regarding to Yazd City. Therefore, Yazd considered as a foci for both *L. major* and *L. tropica* which the first is a zoonotic parasite and the second is an Anthroponotic one. This frequency could affect programming for disease control. Immigrant from rural areas considered as one of the important factors for prevalence of *L. major* and therefore one of the main ways against it would be eradication of rodents. However, the best program against Leishmaniasis results from *L. tropica* would be chemotherapy.

In this study, the majority of lesions were observed on hands. This observation was in agreement of the other studies especially in some other parts of the country (22–24). Appearing of the lesions is often facial in rural areas and on the hands and legs in urban areas (25, 26). That was not the same as our study. We showed that the number of lesions in patients with CL was 1–3. This data was in agreement with other studies in different parts of Iran (27, 28). There is evidence that the most patients appeared more than one lesion and it would be resulted from feeding behavior of the vector with respect.

The prevalence of Leishmaniasis in this area is undoubtedly due to ecological characteristics regarding to vectors and reservoirs. As Leishmaniasis was considered one of the most important diseases from this part of Iran, individual health such as educating, treatment would be appropriate strategies for control and prevention. Moreover, rodent and vector control using rodenticides and insecticides, respectively could reduce the prevalence of this disease in this area.

## Conclusion

The CL found in Yazd Province resulted from *L. major* and *L. tropica* as the agents of rural and urban types, respectively. Around 50% of isolate samples were caused by L. major, and 50% were *L. tropica.* Control programs could be designed for treatment and vector and reservoir control.

## Ethical considerations

Ethical issues (Including plagiarism, informed consent, misconduct, data fabrication and/or falsification, double publication and/or submission, redundancy, etc.) have been completely observed by the authors.
